# Paclitaxel Plus Cetuximab as Induction Chemotherapy for Patients With Locoregionally Advanced Head and Neck Squamous Cell Carcinoma Unfit for Cisplatin-Based Chemotherapy

**DOI:** 10.3389/fonc.2022.953020

**Published:** 2022-07-22

**Authors:** Juan A. Marín-Jiménez, Marc Oliva, Paloma Peinado Martín, Santiago Cabezas-Camarero, Maria Plana Serrahima, Gonzalo Vázquez Masedo, Alicia Lozano Borbalas, María N. Cabrera Martín, Anna Esteve, María C. Iglesias Moreno, Esther Vilajosana Altamis, Lorena Arribas Hortigüela, Miren Taberna Sanz, Pedro Pérez-Segura, Ricard Mesía

**Affiliations:** ^1^ Head and Neck Cancer Unit, Department of Medical Oncology, Catalan Institute of Oncology (ICO – L’Hospitalet de Llobregat), Barcelona, Spain; ^2^ Oncobell Program - Bellvitge Biomedical Research Institute (Institut d'Investigació Biomèdica de Bellvitge), Barcelona, Spain; ^3^ Head and Neck Cancer Unit, Department of Medical Oncology, Hospital Universitario Clínico San Carlos - Instituto de Investigación Sanitaria Hospital Clínica San Carlos, Madrid, Spain; ^4^ Department of Radiation Oncology, Hospital Universitario Clínico San Carlos - Instituto de Investigación Sanitaria Hospital Clínica San Carlos (IdISCC), Madrid, Spain; ^5^ Department of Radiation Oncology, Catalan Institute of Oncology (ICO – L’Hospitalet de Llobregat), Barcelona, Spain; ^6^ Department of Nuclear Medicine, Hospital Universitario Clínico San Carlos - IdISCC, Madrid, Spain; ^7^ Department of Medical Oncology, Catalan Institute of Oncology (ICO-Badalona), B-ARGO group, Institut d’Investigació en Ciències de la Salut Germans Trias i Pujol (IGTP), Barcelona, Spain; ^8^ Oncology Data Analytics Program, Catalan Institute of Oncology (ICO – L’Hospitalet de Llobregat) - Institut d'Investigació Biomèdica de Bellvitge (IDIBELL), Barcelona, Spain; ^9^ Otolaryngology - Head and Neck Surgery Department, Hospital Universitario Clínico San Carlos e- IdISCC, Madrid, Spain; ^10^ Clinical Nutrition Unit, Catalan Institute of Oncology (ICO – L’Hospitalet de Llobregat), Barcelona, Spain

**Keywords:** Head and neck squamous cell carcinoma, head and neck cancer, induction chemotherapy, paclitaxel, cetuximab, radiotherapy, cisplatin, unfit patient

## Abstract

**Objectives:**

Induction chemotherapy (ICT) followed by definitive treatment is an accepted non-surgical approach for locoregionally advanced head and neck squamous cell carcinoma (LA-HNSCC). However, ICT remains a challenge for cisplatin-unfit patients. We evaluated paclitaxel and cetuximab (P-C) as ICT in a cohort of LA-HNSCC patients unfit for cisplatin.

**Materials and Methods:**

This is a retrospective analysis of patients with newly diagnosed LA-HNSCC considered unfit for cisplatin-based chemotherapy (age >70 and/or ECOG≥2 and/or comorbidities) treated with weekly P-C followed by definitive radiotherapy and cetuximab (RT-C) between 2010 and 2017. Toxicity and objective response rate (ORR) to ICT and RT-C were collected. Median overall survival (OS) and progression-free survival (PFS) were estimated using the Kaplan–Meier method. Cox regression analysis was performed to determine baseline predictors of OS and PFS.

**Results:**

A total of 57 patients were included. Grade 3–4 toxicity rate to ICT was 54.4%, and there was a death deemed treatment-related (G5). P-C achieved an ORR of 66.7%, including 12.3% of complete responses (CR). After P-C, 45 patients (78.9%) continued with concomitant RT-C. Twenty-six patients (45.6%) achieved a CR after definitive treatment. With a median follow-up of 21.7 months (range 1.2–94.6), median OS and PFS were 22.9 months and 10.7 months, respectively. The estimated 2-year OS and PFS rates were 48.9% and 33.7%, respectively. Disease stage had a negative impact on OS (stage IVb vs. III–IVa: HR = 2.55 [1.08–6.04], *p* = 0.03), with a trend towards worse PFS (HR = 1.92 [0.91–4.05], *p* = 0.09). Primary tumor in the larynx was associated with improved PFS but not OS (HR = 0.45 [0.22–0.92], *p* = 0.03, and HR = 0.69 [0.32–1.54], *p* = 0.37, respectively).

**Conclusion:**

P-C was a well-tolerated and active ICT regimen in this cohort of LA-HNSCC patients unfit for cisplatin-based chemotherapy. P-C might represent a valid ICT option for unfit patients and may aid patient selection for definitive treatment.

## Introduction

Up to two-thirds of patients with head and neck squamous cell carcinoma (HNSCC) present with locally advanced (LA) disease. At this stage, up to 60% will eventually recur despite curative-intent therapies (1). Treatment usually involves upfront surgery followed by adjuvant (chemo)radiotherapy or definitive chemoradiotherapy (CRT) when organ preservation is preferred or in case of unresectable disease (2–4). In these last two settings, the use of induction chemotherapy (ICT) has been widely debated but it remains a valid option to select patients for larynx-preservation strategy or for those patients with rapidly growing and/or high tumor burden (2, 5, 6).

A significant proportion of patients with newly diagnosed LA-HNSCC are unable to receive cisplatin-based chemotherapy either concurrent to RT or as an ICT regimen. In this scenario, RT with concomitant cetuximab, carboplatin/5-FU, or RT alone with altered fractionation remain the only alternative treatment options (7, 8). Within this group of patients, those with rapidly progressive disease, high tumor volume, or uncontrolled symptoms, as well as those who wish to avoid total laryngectomy, might benefit from an ICT approach.

To date, no prospective randomized trials have evaluated the role of ICT in cisplatin-unfit patients. The PANTERA study (9), a single-arm phase 2 clinical trial, evaluated the combination of paclitaxel plus the anti-EGFR antibody panitumumab as an ICT regimen for patients with LA-HNSCC unfit for cisplatin. Although the trial ended prematurely due to low recruitment and a safety profile worse than expected, two-thirds of patients achieved radiological response by RECIST 1.1, including 8 (15.7%) complete responses (CR). Several retrospective studies have investigated adapted or modified ICT regimens in patients unfit for cisplatin (**Table 1**) (10–13). However, the results obtained in terms of safety and efficacy are difficult to compare given the heterogeneity of patient population and the lack of standard criteria to define frailty and cisplatin unfitness. Paclitaxel in combination with cetuximab, another anti-EGFR antibody, has shown to be a safe and active regimen for patients with recurrent/metastatic disease unfit for cisplatin-based chemotherapy (14). In this setting, the overall response rate (ORR) was 54%, including 22% of CR.

**Table 1:** Summary of available evidence of studies in unfit population for standard ICT.

Study Reference	Study Design	Patient Population	Cisplatin-unfitness/Frailty criteria	ICT regimen	Response to ICT	Post-ICT treatment	Response to radical treatment
**Martínez-Trufero J. et al, 2019** (9)	Prospective Phase 2 study	*N* = 51 Stage III–IVb Unresectable or resectable Mostly larynx and oral cavity	1 of the following: - Age >70 - ECOG 2 - Mild/moderate ACE-27 score - Albumin 2–3.5 g/day	Weekly paclitaxel 80 mg/m^2^ + panitumumab 6 mg/kg q2w.	ORR = 66.7% CRR = 15.7% mPFS = 12.2 months mOS = 31.9 months	RT - panitumumab q2w	CRR = 43.1%
**Patil V.M. et al, 2014** (10)	Retrospective	*N* = 15 Stage IVa–IVb Mostly oral cavity	2 or more criteria: - Age ≥ 60 - ECOG 2/3 - Uncontrolled comorbidity - BMI below 20 kg/m^2^	Weekly paclitaxel 80 mg/m^2^ + CBDCA AUC 2.	ORR = 66.7% CRR = 6.7% mPFS = 10.36 months mOS = 16.53 months	Surgery + CT (*n* = 3) RT-CT (*n* = 3) RT (*n* = 1)	Not reported
**Fayette J. et al, 2016** (11)	Retrospective	*N* = 48 Stage III–IVb Unresectable or resectable Mostly oropharynx and hypopharynx	1 of the following: - Age > 70 - ECOG > 1 - Severe comorbidities - Severe denutrition - Severe toxicity to standard TPF	Docetaxel 40 mg/m^2^, CDDP 40 mg/m^2^, leucovorin 400 mg/m^2^, 5FU bolus 400 mg/m^2^ d1 and 5FU infusion 1000 mg/m^2^/d, d1–2; q2w.	ORR = 83% CRR = 19% mOS = 18.5 months TTR = 22.2 months	Surgery ⟶ RT-CT or RT - cetuximab.	Not reported
**Cochin V. et al, 2018** (12)	Retrospective	*N* = 34 T4N2b-c Unresectable Mostly oropharynx (HPV negative)	Considered unfit for definitive radiotherapy or TPF if: - ECOG ≥2 - Weight loss >10%	CDDP 100 mg/m^2^ d1, 5-FU 1000 mg/m^2^ day 1–4 and cetuximab 400 mg/m^2^ (first dose) - 250 mg/m^2^ d1, 8, 15 q3w.	ORR = 62% mPFS = 5.7 months mOS = 15.5 months	RT-CT (CDDP q3w) or RT - weekly cetuximab.	ORR = 33% CRR = 9%
**Shirashu H. et al, 2020** (13)	Retrospective	*N* = 24 T3-4bN2-3 Unresectable Oropharynx and hypopharynx	Ineligible for TPF as 1 of: - Age ≥ 71 - ECOG 2 - Renal impairment - Cardiac dysfunction - Cerebral infarction - Diabetes	Paclitaxel (60–100 mg/m^2^) d1,8; CBDCA AUC 1.5–2.5 d1,8; cetuximab 400 mg/m^2^ (first dose) - 250 mg/m^2^ d1, 8, 15 q3w.	ORR = 87% CRR = 4% mPFS = 29.4 months mOS = 34.8 months	RT-CT (CDDP q3w or CBDCA q3w or CBDCA q1w) or RT - weekly cetuximab	ORR = 88%

T, tumor stage; N, lymph node stage; ECOG, Eastern Cooperative Oncology Group performance status; ACE-27, Adult Comorbidity Evaluation-27; BMI, body mass index; CBDCA, carboplatin; CDDP, cisplatin; 5-FU, 5-fluorouracil; TTR, time to relapse; NR, not reached.

The aim of this study was to evaluate the tolerance and antitumor activity of P-C as an ICT regimen in a retrospective cohort of patients with newly diagnosed LA-HNSCC unfit for cisplatin-based chemotherapy.

## Patients And Methods

### Study Population and Design

A retrospective cohort analysis of newly diagnosed LA-HNSCC patients treated with paclitaxel in combination with cetuximab as ICT regimen between January 2010 and December 2017 was performed in two Spanish institutions—Institut Català d’Oncologia (L´Hospitalet, Barcelona) and Hospital Clínico San Carlos (Madrid). Selection criteria for inclusion in the analysis were as follows: (1) cytologically and/or histologically confirmed HNSCC from the oral cavity, oropharynx, hypopharynx, or larynx, or cervical squamous cell carcinoma with unknown primary; (2) stage III–IVb disease with no evidence of distant metastases (M0) according to the 7th edition TNM; (3) patient ineligibility for cisplatin-based chemotherapy due to age ≥70 years old and/or Eastern Cooperative Oncology Group performance status (ECOG) ≥2 and/or significant comorbidity; and (4) received ICT with P-C (at least one dose) in the context of (a) indication for an upfront-chemotherapy strategy (highly symptomatic and/or rapidly progressive and/or technically unresectable disease or inoperable patient), based on medical oncologist and ENT surgeon evaluation and after consensus at the Multidisciplinary Tumor Board (MTB); and (b) patient preference for an organ-preservation strategy to avoid total laryngectomy. Patient demographics, disease characteristics, treatment delivery, tumor response, and toxicity (grade 3–4 adverse events (AEs) according to v4.0 National Cancer Institute common terminology criteria for AEs (NCI-CTCAE) were retrospectively reviewed by two independent investigators. Charlson comorbidity index (CCI) (15) was retrospectively calculated for each patient according to reported comorbidities in the electronic medical records.

### Treatment and Follow-Up Assessment

Treatment schedule, evaluation of response, and follow-up assessments were conducted as per institutional protocols. P-C was administered weekly (paclitaxel 80 mg/m^2^ and cetuximab loading dose of 400 mg/m^2^ followed by 250 mg/m^2^, both intravenously) for a total of 9 weeks, although treatment continued beyond 9 weeks until RT initiation in selected cases to avoid periods of time without treatment. Radiological response evaluation was performed according to RECIST 1.1. Patients with radiological response (CR or partial response, PR) or stable disease (SD) and clinical benefit after ICT were planned for sequential treatment with RT-C. In both institutions, controversial cases were discussed by treating oncologists and decided by consensus with other medical teams at MTB. Patients considered ineligible for concurrent RT-C treatment were offered definitive RT alone, or best supportive care (BSC). RT-C was administered as per standard of care according to institutional protocols (IMRT 70 Gy in 35 fractions at 2 Gy/fraction plus weekly cetuximab at 250 mg/m^2^). Response evaluation was performed with a head and neck CT scan at 8–10 weeks and/or PET/CT scan at 12 weeks from the last RT dose as per institutional guidelines. After treatment completion, follow-up was performed according to institutional protocols (16). Locoregional recurrences were confirmed histologically when feasible, and distant metastases were diagnosed by unequivocal clinical/radiological evidence. Survival status was determined by July 1, 2021, which was the data cutoff for the analysis.

### Statistical Analysis

ORR and CR rate (CRR) after ICT and at completion of definitive RT were calculated. OS and PFS from the date of ICT initiation were estimated using the Kaplan–Meier method. Patients who were lost to follow-up or were still alive without recurrence by the end of the study were censored at the date of last follow-up. OS, PFS, and relapse-free survival (RFS) rates were also estimated. Comparisons between groups were performed using the log-rank test. Cox proportional hazards regression model was performed to explore potential predictors of OS and PFS among baseline characteristics including gender (male vs. female), age (<70 vs. ≥70), tobacco exposure (never/former vs. active smoker), alcohol intake (non/moderate vs. heavy use, ECOG (0/1 vs. 2), tumor location (larynx vs. other), tumor stage (III/IVa vs. IVb), CCI (score <7 vs. ≥7), and baseline comorbidities. Data analysis and graphs were performed using IBM SPSS Statistics v19.0 for windows and GraphPad Prism v9.1.1 for macOS. Univariate and multivariate Cox regression analysis for OS and PFS were performed using the *survival* package (17) in RStudio Version 1.4.1106 for macOS. Hazard ratios (HR) and 95% confidence intervals (CI) are reported. Significant (*p* < 0.05) predictors in the univariate analysis along with gender and age were included in the multivariate analysis (**Supplementary Table 1**).

## Results

### Cohort Characteristics

**Figure 1:**
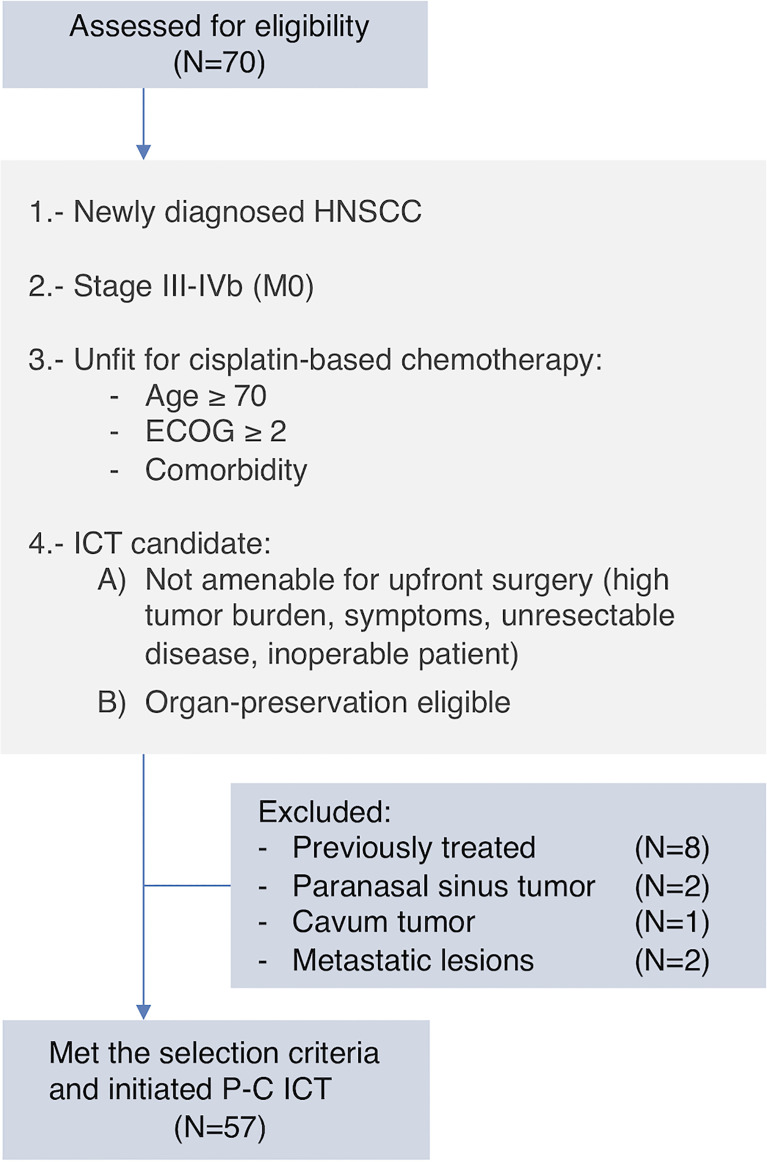
Flowchart of patient selection and inclusion criteria.

A total of 57 patients were included and deemed eligible for the analysis (**Figure 1**). Baseline characteristics are summarized in **Table 2**. Most patients were male (91.2%) and ≥70 years old (70.2%), and had stage IV disease (80.7%) and an ECOG performance status ≤1 (78.9%). Half of the patients had ≥2 cisplatin ineligibility criteria. Larynx (35.1%) was the most frequent primary tumor location, followed by oropharynx (22.8%) and oral cavity (19.3%). Cardiovascular, pulmonary, and peripheral vascular disease were the three most prevalent comorbidities, and CCI median score was 7 points for the entire cohort.

**Table 2:** Baseline patient and tumor characteristics of the whole cohort.

Variable	Total (*n* = 57)
**Age**	
Median (range), years ≥70 years old, *n* (%)	74 (48–88) 40 (70.2)
**Gender**, *n* (%)	
Female Male	5 (8.8) 52 (91.2)
**ECOG**, *n* (%)	
0 1 2	4 (7) 41 (71.9) 12 (21.1)
**Tobacco status**, *n* (%)	
Never smoker Former smoker Active smoker Unknown	9 (15.8) 32 (56.1) 13 (22.8) 3 (5.3)
**Tobacco lifetime exposure**	
Pack-years, mean (range)	59 (15–135)
Alcohol intake*, *n* (%)	
None Moderate Heavy Unknown	22 (38.6) 12 (21.1) 19 (33.3) 4 (7.0)
**Type of comorbidity**, *n* (%)	
Pulmonary Cardiovascular Peripheral vasculopathy Nephropathy Hepatopathy Central neuropathy Peripheral neuropathy	17 (29.8) 17 (29.8) 14 (24.6) 7 (12.3) 7 (12.3) 3 (5.3) 2 (3.5)
**Charlson comorbidity index**	
Score, mean (range) ≥7 points, *n* (%)	7 (3–14) 34 (59.6)
**Cisplatin ineligibility criteria**, *n* (%)	
Age ≥ 70 years old ECOG = 2 Presence of comorbidity contraindicating cisplatin	40 (70.2) 12 (21.1) 34 (59.6)
**Number of met cisplatin ineligibility criteria per patient**, *n* (%)	
1 2 3 Not specified	23 (40.4) 24 (42) 5 (8.8) 5 (8.8)
**Primary tumor location**, *n* (%)	
Larynx Oropharynx HPV positive HPV negative Unknown Oral cavity Hypopharynx Unknown primary location	20 (35.1) 13 (22.8) 2 2 9 11 (19.3) 7 (12.3) 6 (10.5)
**Tumor stage** (TNM 7th edition), *n* (%)	
III IVa IVb	11 (19.3) 34 (59.6) 12 (21.1)

*Alcohol intake categories were defined according to the number of standard units of alcohol per week as follows: moderate: male patients <21, female patients <14; heavy: male patients ≥21, female patients ≥14. ECOG, Eastern Cooperative Oncology Group performance status.

### ICT Treatment

#### Compliance and Toxicity

A total of 30 patients (52.6%) completed 9 cycles of P-C as planned. The median number of P and C administered cycles were 8 and 9 (both ranging 2–14), respectively. The average duration of ICT was 8.8 weeks (95% CI 7.7–9.9). Twenty-two patients (38.6%) required dose reduction of P (21 [36.8%] patients) and/or C (19 [33.3%] patients). Overall, 26 patients (45.6%) and 31 patients (54.4%) presented grade 1–2 and grade 3–4 toxicity to P-C, respectively (**Table 3**). One patient died due to respiratory failure secondary to a pneumonitis in the context of CMV infection and G3 neutropenia related to paclitaxel. The treating physician and respirologist felt that the pneumonitis was likely related to CMV although the potential contribution of paclitaxel and/or cetuximab could not be completely ruled out, and thus, it was finally deemed as possibly related.

**Table 3:** Relevant AEs (grade 3–5) to ICT and radical phase of treatment.

Toxicity	ICT (*n* = 57)			RT-C (*n* = 48)	
	Grade 3*n* (%)	Grade 4*n* (%)	Grade 5*n* (%)	Grade 3*n* (%)	Grade 4*n* (%)
Hematological					
Anemia Neutropenia	2 (3.5) 4 (7)	0 1 (1.8)	0 0	1 (2.1) 0	0 0
Gastrointestinal					
Diarrhea Esophagitis	2 (3.5) 1 (1.8)	0 0	0 0	0 0	0 0
Oral mucositis	2 (3.5)	0	0	20 (41.7)	0
Skin toxicity	20 (35.1)	0	0	17 (35.4)	2 (4.7)
Asthenia	2 (3.5)	0	0	0	0
Anorexia	2 (3.5)	0	0	1 (2.1)	0
Pneumonitis	0	0	1 (1.8)	0	0
Peripheral neuropathy	0	1 (1.8)	0	0	0
Ocular toxicity	2 (3.5)	0	0	0	0
Infusional reaction	1 (1.8)	0	0	0	0
Hypomagnesemia	1 (1.8)	1 (1.8)	0	1 (2.1)	0
Other	3 (5.3)	0	0	0	0

#### Treatment Response

**Figure 2:**
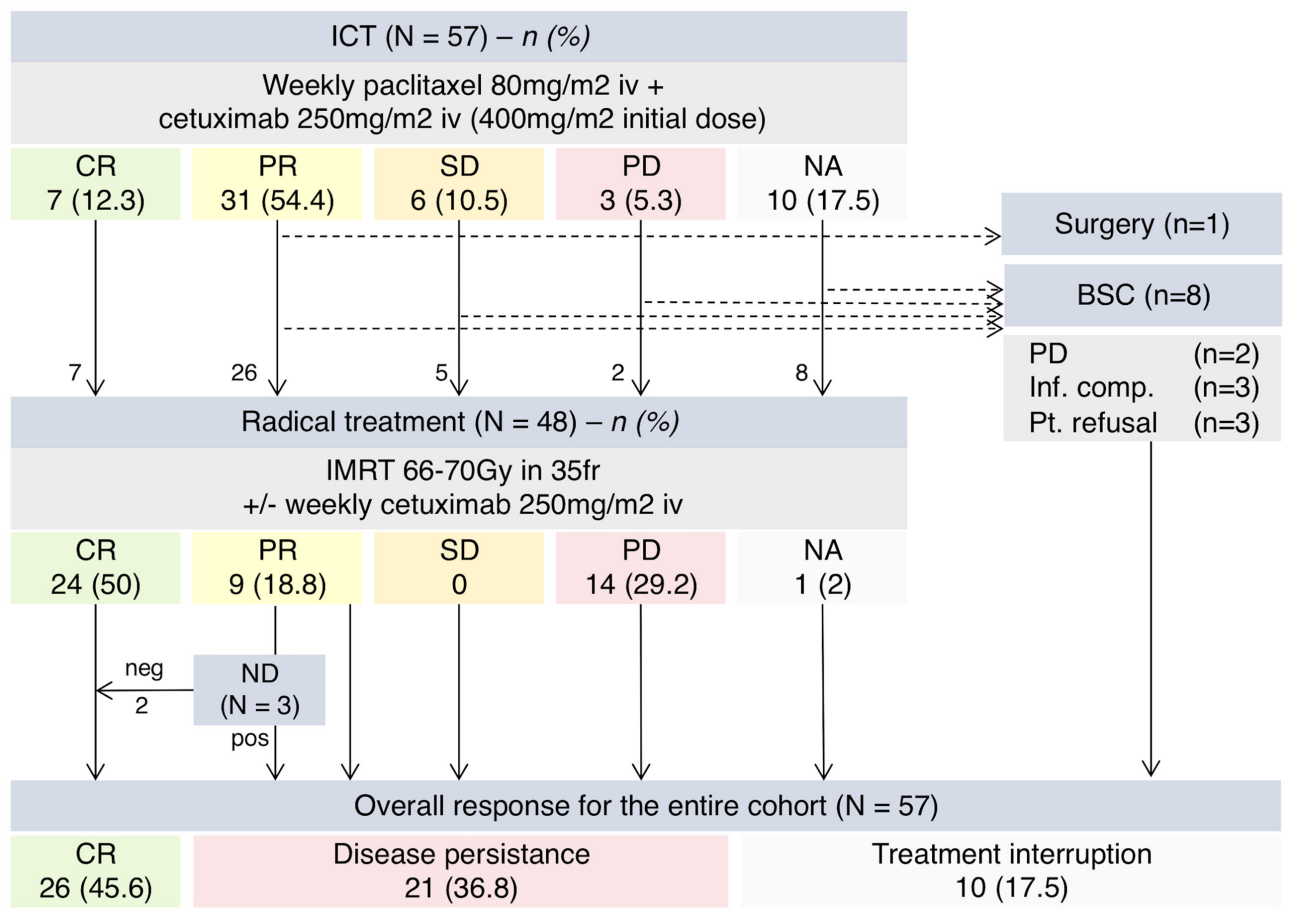
Schema summarizing treatment received and ORR. ICT, induction chemotherapy; ND, neck dissection; CR, complete response; PR, partial response; SD, stable disease; PD, progressive disease; NA, not available; BSC, best supportive care; Inf. Comp., infectious complications; Pt., patient.

Tumor radiological evaluation was performed in 47 patients (82.5%): 7 (14.9%) achieved a CR, 31 (66.0%) achieved PR, 6 (12.8%) had SD, and 3 patients (6.4%) had PD. The ORR to ICT was 66.7% (38 of 57 patients) and CRR was 12.3% (**Figure 2**). Of those 10 patients who were not radiologically assessed, two died before completing the ICT due to disease progression and respiratory infection, respectively. The remaining 8 patients had clinical response or stability according to treating physicians.

### Radical Treatment

Following ICT, 48 (84.2%) patients underwent definitive RT: 45 received RT-C and 3 received RT alone due to prior intolerance/toxicity to cetuximab during ICT. One patient underwent surgery instead of RT-C after achieving PR to ICT. Eight patients (14.0%) discontinued treatment due to toxic death (*n* = 1), PD (*n* = 2), infectious complications (*n* = 2), and patient refusal (*n* = 3) (**Figure 2**).

#### Compliance and Toxicity

RT completion rate was 87.5%. Six patients (12.5%) could not complete RT due to severe oral and/or skin toxicity (*n* = 5) and respiratory infection (*n* = 1). Overall, 19 (39.6%) and 29 (60.4%) patients experienced grade 1–2 and grade 3–4 toxicity, respectively (**Table 3**).

#### Treatment Response

Of those patients treated with definitive RT, 24 patients (50%) achieved a CR, 9 had (18.8%) PR, and 14 (29.2%) had PD. One patient died before response evaluation due to bilateral pneumonia unrelated to treatment. Due to equivocal response, eight patients were evaluated both with CT-scan first and then PET-CT at 12 weeks post-RT: 4 patients showed persistent disease, 2 patients achieved CR, and 2 patients were diagnosed with distant metastases. Three patients with PR due to suspected nodal disease persistence but CR of the primary tumor underwent ND upon MTB evaluation. Pathology was negative for malignancy in two patients, who were considered complete responders in the final analysis. Overall, CRR after radical treatment was 45.6% (26 of 57 patients) (**Figure 2**).

### Outcomes

**Figure 3:**
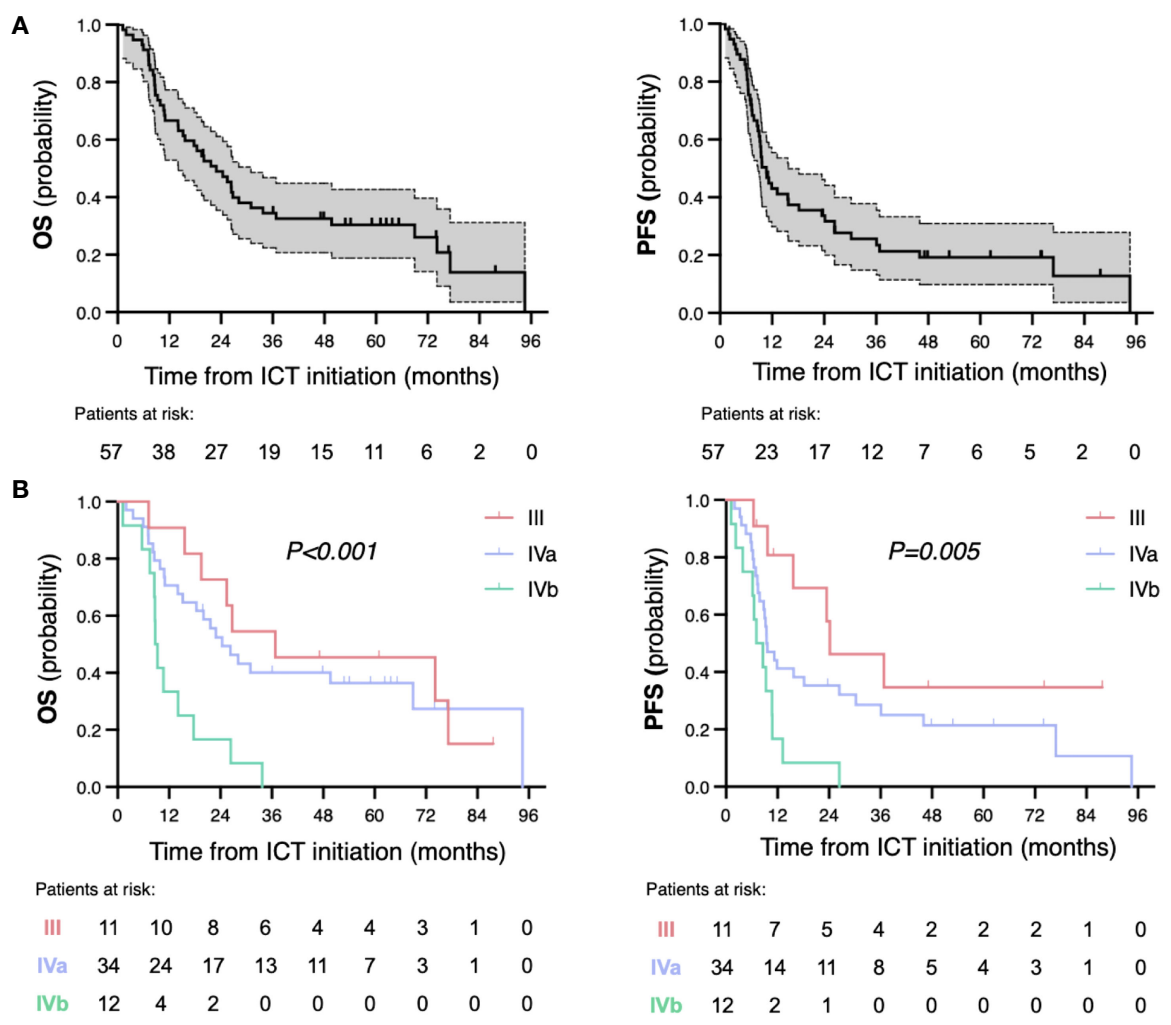
Kaplan–Meier survival curves for overall survival (OS) and progression-free survival (PFS) of the whole cohort **(A)** and by tumor stages **(B)**. *p* represents log-rank *p*-value for subgroup comparison. ICT, induction chemotherapy.

The median follow-up of the whole cohort was 21.7 months (range 1.2–94.6). Median OS and PFS were 22.9 months (95% CI 14.7–31.1) and 10.7 months (95% CI 8.5–12.9), respectively (**Figure 3A**). One-, 2-, and 3-year survival rates were 66.7%, 48.9%, and 34.4% for OS and 43%, 33.7%, and 25.6% for PFS. Median OS and PFS significantly differed by tumor stage (III vs. IVa vs. IVb): 36.8 vs. 24.4 vs. 8.8 months (*p* = 0.001) and 24.2 vs. 9.5 vs. 7.1 months (*p* = 0.005), respectively (**Figure 3B**). Among patients who achieved CR, 1- and 2-year RFS rate were 92.6% and 81.5%, respectively. By the time of data cutoff, 8 patients (30.8%) had recurred: 3 patients with locoregional disease and 5 with distant metastases.

In the multivariate analyses for OS, stage IVb was significantly associated with worse OS (HR = 2.55, [1.08–6.04], *p* = 0.03), with a trend towards worse PFS (HR = 1.92 [0.91–4.05], *p* = 0.09) (**Table 4**). Primary tumor in the larynx was associated with improved PFS but not OS (HR = 0.45 [0.22–0.92], *p* = 0.03, and HR = 0.69 [0.32–1.54], *p* = 0.37, respectively). The absence of peripheral neuropathy was also significantly associated to longer PFS, likely due to decompensated categories.

**Table 4:** Multivariate Cox regression analysis for OS and PFS.

Variable		*n*	OS		PFS	
			HR (95% CI)	P	HR (95% CI)	P
**Gender**	Male Female	52 5	1 1.75 (0.59–5.19)	0.31	1 1.25 (0.43–3.62)	0.68
**Age**	<70 ≥70	17 40	1 1.46 (0.70–3.03)	0.31	1 1.07 (0.56–2.07)	0.83
**Tobacco status**	Never/former Active	41 13	1 1.73 (0.80–3.78)	0.17	Not entered	–
**Peripheral neuropathy**	No Yes	55 2	1 3.50 (0.72–17.08)	0.12	1 7.22 (1.41–37.02)	**0.02**
**Primary tumor location**	Other Larynx	37 20	1 0.69 (0.32–1.54)	0.37	1 0.45 (0.22–0.92)	**0.03**
**Tumor stage**	III/IVa IVb	45 12	1 2.55 (1.08–6.04)	**0.03**	1 1.92 (0.91–4.05)	0.09

Entered predictors were selected according to the univariate analysis for OS and PFS (see **Supplementary Table 1**). Significant values (p<0.05) are marked in bold. Likelihood ratios for the multivariate analysis models were 15.85 (*p* = 0.01) for OS and 16.76 (*p* < 0.01) for PFS. HR, hazard ratio; ECOG, Eastern Cooperative Oncology Group performance status; com, comorbidity; CCI, Charlson comorbidity index.

### Laryngeal Cancer Subgroup Analysis

**Figure 4:**
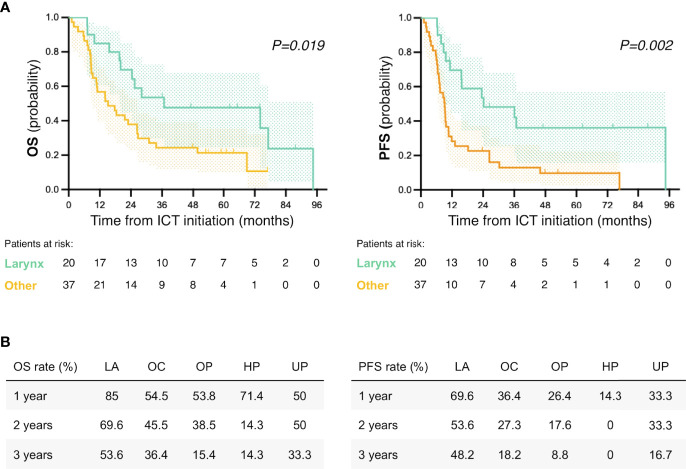
Survival analysis according to primary tumor location. **(A)** Kaplan–Meier survival curves for overall survival (OS) and progression-free survival (PFS) of larynx vs. other primary tumor locations. *p* represents log-rank *p*-value for subgroup comparison. **(B)** OS and PFS rates by primary tumor location for the 1st, 2nd and 3rd year after ICT initiation. ICT, induction chemotherapy; LA, larynx; OC, oral cavity; OP, oropharynx; HP, hypopharynx; UP, unknown primary.

In the subgroup of patients with laryngeal cancer (*n* = 20), the mean age was 72.1 years and mean CCI score was 8. All patients had stage III or IVA at diagnosis (9 and 11 patients, respectively). The ORR to ICT was 80% (16 of 20) including 3 (15%) CR. After ICT, all patients continued with radical treatment. Nineteen patients (95%) were treated with RT-C and one patient received RT alone due to prior toxicity to cetuximab. Sixteen patients (80%) achieved CR, including one patient with suspected persistent disease according to PET-CT evaluation who underwent ND and biopsy of a residual lesion, both with negative pathological result. At the time of data cutoff, 1 patient recurred locoregionally and 3 distantly. Twelve patients (60%) had a preserved functional larynx (no tracheostomy, voice prosthesis, or any other procedure/intervention due to laryngeal malfunction) at the last follow-up. With a median follow-up of 32 months (range 7.1–94.6), median OS and PFS were 36.8 months (95% CI 0–84.4) and 24.2 months (0–51.5), respectively (**Figure 4A**). The estimated 1-, 2-, and 3-year OS and PFS rates were also higher for the larynx subgroup (**Figure 4B**).

## Discussion

In this retrospective analysis, P-C was an active ICT regimen for patients with LA-HNSCC unfit for cisplatin, achieving an ORR and CRR of 66.7% and 12.3%, respectively. Although up to 54.4% patients experienced at least one grade 3–4 AE related to P-C, toxicity was manageable; 52.6% patients completed 9 cycles of treatment and up to 85% continued with definitive RT. Following definitive RT, almost 50% of patients achieved CR, with a 2-year RFS rate of 81.5%. Median OS and PFS for the whole cohort were 22.9 months and 10.7 months, respectively.

Patients with LA-HNSCC unfit for cisplatin are under-represented in clinical trials, particularly in those involving the evaluation of ICT strategies. TPF is currently the regimen of choice when considering an ICT approach for patients who are fit for cisplatin (17, 18), with expected ORR of up to 70% in both resectable and unresectable LA-HNSCC (18–20). Despite evaluating a much more fragile cohort of patients, the observed response rates of P-C in our cohort were at least comparable. In this regard, the PANTERA phase 2 trial showed an almost identical ORR in a more similar yet selected patient population (9, 21). These efficacy results were also in line with other retrospective series evaluating adapted ICT regimens in this group of patients (see **Table 1**). Moreover, beyond tumor response, improving patient selection for organ preservation and ensuring sequential treatment with definitive RT are also factors to be considered when evaluating ICT. The percentage of patients continuing to definitive RT after TPF ICT ranged between 68% and 91% according to trials (5, 22–24). In the PANTERA trial, 41 of 51 patients (80.4%) proceeded to definitive RT following panitumumab-paclitaxel (9). In line with these results, in our cohort, up to 84.2% were eligible for sequential treatment after P-C. However, it should be noted that no prospectively pre-specified criteria were applied for the decision-making process in our cohort. If radiological response or stable disease had been required, the percentage of patients proceeding to definitive RT would have dropped to 66.7% in our study (see **Figure 2**).

In addition to efficacy, one of the most debated aspects of ICT is the rate of AEs and the potential compromise of definitive radiotherapy. Randomized trials evaluating the TPF regimen had shown significant toxicity (i.e., grade 3 and 4 febrile neutropenia ranging between 19% and 36%) and worrying mortality rates (up to 6%) (25) despite including a fit and selected patient population. The toxicity was also unexpectedly high in adjusted regimens such as paclitaxel-panitumumab, with grade 3–4 AEs occurring in 73% of the patients (9). However, the AEs spectrum of the anti-EGFR and paclitaxel combination is not comparable to that for TPF as grade 3–4 AEs mainly involved skin toxicity and mucositis and only led to treatment discontinuation in 11.8% of patients (9). In our cohort, grade 3 and 4 toxicity rate to P-C (54.4%) was lower than the combination with panitumumab, probably due to a much lower rate of severe mucositis (3.5% vs. 19.6%). Of note, a fatal treatment-related event did occur in our cohort.

Proper patient selection remains one of the main challenges for clinicians in daily practice. Criteria to define unfitness for cisplatin and identify those patients at high risk of toxicity have been proposed (26–28), and should be considered in prospective studies involving this group of patients. In addition, the criteria used for treatment decisions in standard-of-care practice should be well reflected in electronic medical history in order to improve real-world-data studies such as the present analysis. Age and comorbidity are well-known factors affecting treatment response and prognosis (29–32), but they are not recommended to independently guide treatment decision-making process anymore. Comprehensive geriatric assessment (CGA) and a multidisciplinary assessment by the MTB have been established as the standard for elderly and fragile patients with LA-HNSCC (33, 34). Unfortunately, at the time our cohort was treated, CGA was not yet implemented in clinical practice in our setting, so a thorough evaluation of patients´ fitness is missing in our study and would have helped to improve patient selection process.

In our cohort, patients with laryngeal tumors seemed to benefit to a greater extent from P-C ICT, as 80% of them achieved a CR and just one patient had locoregional recurrence by the time of data cutoff. It is well-known that patients with laryngeal cancer have overall better prognosis when compared to other tumor locations. Most of the laryngeal cancer patients (17/20) in our cohort were deemed eligible for an organ-preservation strategy, while the other three patients were considered inoperable. This, and the absence of stage IVb disease in this patient subgroup, may explain the better performance of this subgroup. However, and despite the limited number of patients, we believe that P-C may help in selecting patients for a radical treatment and might be a reasonable larynx preservation approach in the cisplatin-unfit patient population.

We acknowledge the limitations inherent to the retrospective nature of our analysis and the small sample size of the cohort. Oncologic outcomes and toxicity rates were collected retrospectively from electronic clinical records, and potential confounders might have biased the analysis. The impact on quality of life, incidence of complications, and rate of related hospitalizations were not available and should be included in prospective studies considering the poor general condition of this patient population. The study was not powered to detect differences in specific subsets, and the heterogeneous study population might have impacted our results. No predefined criteria were followed for treatment decision-making; therefore, potential differences among the two centers cannot be ruled out. Of note, the decision of radical treatment was not subjected to predefined response criteria or RECIST, but made according to treating clinicians’ evaluation. Additional patient-centered efficacy measures, such as quality of life and patient-reported outcomes, are also lacking, and should be considered when evaluating ICT regimens.

In conclusion, the results from our study suggest that the P-C appears to be a tolerable and active regimen for patients with LA-HNSCC who are unfit for cisplatin-based chemotherapy but are eligible for ICT. Those patients with laryngeal primaries achieved the longest survival, encouraging further evaluation of P-C as an organ-preservation strategy in a clinical trial.

## Data Availability Statement

The original contributions presented in the study are included in the article. Further inquiries can be directed to the corresponding authors.

## Author Contributions

The authors confirm contribution to the paper as follows: study conception and design: M-JJA, OM, C-CS, TM, and MR; data collection: M-JJA, PMP, and C-CS; analysis and interpretation of results: M-JJA, OM, PMP, C-CS, EA, TM, and MR; draft manuscript preparation: M-JA and OM; manuscript editing: M-JJA, OM, PM, LA, TM, and MR. All authors reviewed the results and approved the final version of the manuscript. M-JA and OM have contributed equally to this work and share first authorship. P-SP and MR share senior authorship. All authors contributed to the article and approved the submitted version.

## Conflict of Interest

OM: Advisory Role: Merck and Bristol Myers Squibb. Financial Interests, Personal, Other, Travel/Accommodation expenses: MSD Oncology, Merck, Bristol Myers Squibb, and Transgene. Personal and/or Institutional Research Grant: GlaxoSmithKline and Roche. Financial Interests, Personal, and/or Institutional Research Grant: Bristol-Myers Squibb, Merck, MSD Oncology, Isa Therapeutics, AXL Oncology, Boehringer Ingelheim, Roche, Debiopharm, Abbvie, and Ayala Therapeutics. C-CS reports advisory role for Merck KGaA and Bristol-Myers Squibb; grant/research support (clinical trials) from AstraZeneca, Merck Sharp & Dohme, Pfizer, and Merck KGaA; travel and academic expenses from Merck KGaA, Bristol-Myers Squibb, and Merck Sharp & Dohme. PM reports consultant role for Nanobiotix and travel non-financial support and academic work fees from Merck, Eisai, and Bristol-Myers Squibb. TM reports consultant role for MSD, Merck, and Nanobiotics; speaker’s bureau for Bristol-Myers Squibb, MSD, AstraZeneca, and Merck; and travel and academic work fees from Merck and MSD. MR reports consultant role for BMS, MSD, Merck, Astra Zeneca, Nanobiotics, Roche, and Bayer; speaker’s bureau for BMS, MSD, Roche, and Merck; and travel and academic work fees from Roche, BMS, and Merck.

The remaining author declares that the research was conducted in the absence of any commercial or financial relationships that could be construed as a potential conflict of interest.

## Publisher’s Note

All claims expressed in this article are solely those of the authors and do not necessarily represent those of their affiliated organizations, or those of the publisher, the editors and the reviewers. Any product that may be evaluated in this article, or claim that may be made by its manufacturer, is not guaranteed or endorsed by the publisher.

## Supplementary Material

Supplementary Table 1 Univariate Cox regression analysis for OS and PFS. Selected variables for the multivariate analysis are marked in bold. HR, hazard ratio; ECOG, Eastern Cooperative Oncology Group performance status; com, comorbidity; CCI, Charlson comorbidity index.
