# Supplementary material for: Paclitaxel Plus Cetuximab as Induction Chemotherapy for Patients With Locoregionally Advanced Head and Neck Squamous Cell Carcinoma Unfit for Cisplatin-Based Chemotherapy
Source: Front Oncol. 2022 Jul 22;12:953020. doi: 10.3389/fonc.2022.953020 (PMC9355730; doi:10.3389/fonc.2022.953020)
Supplement: Supplementary Table 1 — Univariate Cox regression analysis for OS and PFS. Selected variables for the multivariate analysis are marked in bold. HR, hazard ratio; ECOG, Eastern Cooperative Oncology Group performance status; com, comorbidity; CCI, Charlson comorbidity index. [file Table_1.docx]

**Supplementary Table 1.** **Univariate Cox regression analysis for OS and PFS**. Selected variables for the multivariate analysis are marked in bold.

| **VARIABLE** | | ***n*** | **OS** | | **PFS** | |
| --- | --- | --- | --- | --- | --- | --- |
|  |  |  | HR (CI 95%) | *P* | HR (CI 95%) | *P* |
| **Gender** | Male  Female | 52  5 | 1  1.30 (0.46-3.67) | **0.62** | 1  1.08 (0.38-3.05) | **0.88** |
| **Age** | <70  ≥70 | 17  40 | 1  1.11 (0.55-2.21) | **0.78** | 1  0.97 (0.51-1.85) | **0.92** |
| **Tobacco status** | Never/former  Active | 41  13 | 1  2.43 (1.22-4.85) | **0.01** | 1  1.69 (0.85-3.37) | 0.14 |
| **Alcohol intake** | None/moderate  Heavy | 34  19 | 1  1.19 (0.62-2.25) | 0.61 | 1  1.10 (0.59-2.06) | 0.77 |
| **ECOG** | 0/1  2 | 45  12 | 1  0.98 (0.45-2.12) | 0.95 | 1  1.07 (0.56-2.16) | 0.86 |
| **Cardiovascular com.** | No  Yes | 40  17 | 1  0.92 (0.47-1.79) | 0.80 | 1  0.55 (0.27-1.11) | 0.10 |
| **Pulmonary com.** | No  Yes | 40  17 | 1  1.37 (0.70-2.66) | 0.36 | 1  1.38 (0.73-2.58) | 0.32 |
| **Hepatopathy** | No  Yes | 50  7 | 1  1.74 (0.73-4.15) | 0.22 | 1  1.37 (0.57-3.26) | 0.48 |
| **Nephropathy** | No  Yes | 50  7 | 1  1.64 (0.73-3.72) | 0.23 | 1  0.98 (0.39-2.49) | 0.97 |
| **Central neuropathy** | No  Yes | 54  3 | 1  1.78 (0.43-7.41) | 0.43 | 1  2.00 (0.61-6.52) | 0.25 |
| **Peripheral**  **vasculopathy** | No  Yes | 43  14 | 1  1.00 (0.43-2.05) | 0.99 | 1  0.83 (0.40-1.72) | 0.61 |
| **Peripheral**  **neuropathy** | No  Yes | 55  2 | 1  3.88 (0.90-16.66) | **0.07** | 1  10.1 (2.01-50.29) | **<0.01** |
| **CCI** | <7 score  ≥7 score | 18  39 | 1  0.93 (0.48-1.77) | 0.82 | 1  0.82 (0.44-1.54) | 0.54 |
| **Primary tumor location** | Other  Larynx | 37  20 | 1  0.44 (0.22-0.89) | **0.02** | 1  0.37 (0.19-0.71) | **<0.01** |
| **Tumor stage** | III/IVa  IVb | 45  12 | 1  3.61 (1.77-7.34) | **<0.01** | 1  2.76 (1.39-5.52) | **<0.01** |
| Abbreviations: HR = hazard ratio, ECOG = Eastern Cooperative Group performance status, com = comorbidity, CCI = Charlson comorbidity index. | | | | | | |
